# Crystal structure prediction: achievements and opportunities

**DOI:** 10.1107/S2052252523001835

**Published:** 2023-03-01

**Authors:** C. Richard A. Catlow

**Affiliations:** a Department of Chemistry, University College London, 20 Gordon Street, London WC1H 0AJ, United Kingdom; b School of Chemistry, Cardiff University, Park Place, Cardiff CF10 3AT, United Kingdom

**Keywords:** structural science, computational modelling of crystal structures, crystal structure prediction, nano-particle structures, machine learning, editorial

## Abstract

This editorial gives gives an update on the current status of crystal structure prediction and its opportunities and challenges.

The role of computational modelling in structural science was discussed in an Editorial in 2020 (Catlow, 2020 ▸). The field is, however, evolving rapidly and it is timely to return to the theme of Structure Prediction, starting with the celebrated quotation from the News and Views article of John Maddox, Editor of *Nature*, published in 1988:

One of the continuing scandals in the physical sciences is that it remains impossible to predict the structure of even the simplest crystalline solids from a knowledge of their composition.

The statement was of questionable accuracy but provided a powerful stimulus to the growing field of crystal structure modelling and prediction, with a response by the present author and Price, published in *Nature* two years later (Catlow & Price, 1990 ▸). The field was then reviewed twenty years after Maddox’s article (Woodley & Catlow, 2008 ▸), and more recently by Oganov (2018 ▸) and Woodley *et al.* (2020 ▸). But how far has the community responded to the ‘Maddox Challenge’ over thirty years after it was first issued?

It is first necessary to recall the distinction between ‘modelling’ and ‘prediction’. The ability to model crystal structures has a long history including the early work of Pauling. Then in the 1980s, it became clear that relatively simple interatomic potential models coupled with energy minimization procedures could model accurately the structures of a wide range of solids including silicates (Catlow *et al.*, 1982 ▸) and zeolites (Henson *et al.*, 1994 ▸); similar success was enjoyed in modelling molecular crystals. However, this was not prediction: it showed that transferable potentials would generate an energy minimum structure that was close to that experimentally observed; and this capability could help in refining structures. But prediction requires a search of the configurational space spanned by the structural variables to identify regions, which may correspond to stable structures, and which can then be refined by minimization of the energy calculated either using an interatomic potential model or using quantum mechanical techniques.

The exploration of energy landscapes is a major field of theoretical and computational science with crystal structure prediction being one key area. Notable progress was made in structure prediction for inorganic materials by Jansen and Schön who explored energy landscapes using simulated annealing techniques, reviewed by Schön & Jansen (2009 ▸). In a series of studies, Woodley and co-workers have shown the power of Genetic Algorithm methods in structure prediction (Lazauskas *et al.*, 2017 ▸). Successful predictions have been made in several cases by the simple expedient of generating a large number of random configurations which are then subjected to energy minimization with the resulting lowest energy structure being the predicted structure [see, for example, Pickard & Needs (2011 ▸)]. The approach is compared with search methods by Woodley & Sokol (2012 ▸).

Different types of crystal structure may need different methods. For network and framework structures, topological principles may be used – an approach that has a long history going back to the early work of Wells (1954 ▸). It has enjoyed considerable success in the field of structure prediction in microporous materials as illustrated by Bell, Klinowski, Treacy and co-workers [see, for example, Foster *et al.* (2004 ▸) and Treacy *et al.* (2004 ▸)]. While the very active area of molecular crystal structure prediction explores structural possibilities using packing algorithms. Recent developments in the field are reviewed by Price (2018 ▸), with the prediction of the structures of polymorphs of pharmaceutical compounds being of particular interest and importance.

This editorial cannot do justice to a large and increasingly diverse field and the interested reader should consult the articles cited and references therein. But what is the current status of the field and where are the opportunities and challenges?

Structure prediction methods can, of course, be extended to other areas of structural science notably nano-particle structures where recent illustrations include the work of Escatllar *et al.* (2019 ▸) using GA methods coupled with DFT refinement, which predicted the structures of magnesium silicate nano-particles of relevance to the study of the chemistry of cosmic dust grains.

The field continues to benefit from methodological developments. A major limitation of most methods used for CSP is the lack of information that they provide about the global structure of the lattice energy landscape: the pathways and energy barriers between predicted structures, and how structures group into superbasins. A recent article by Yang & Day (2022 ▸) analysing energy landscapes of molecular crystals applied a Monte Carlo approach to approximate energy barriers between crystal structures of known polymorphic molecules, showing how the results help understand the kinetic stability of high-energy structures.

Another interesting technical development is reported by Dickson *et al.* (2022 ▸) who examined systems with variable composition and were able to implement variable compositions within the runs while searching a space of compositions.

A further fascinating recent development is the work of Price & Price (2022 ▸), which offers insight into the sensitivity of organic crystal packing to the substituents, based on an analysis of 232 crystal structures of ‘chalcones’ with only one small substituent on each phenyl ring. Although most of each molecule was the (chalcone) molecular scaffold, remarkably there were over 170 different packings. The only large isostructural group was of 15 molecules with all substituents, mainly halogens, in the *para* position. How can such structural diversity be compatible with the molecules all having the same chalcone scaffold? This diversity is closely mirrored by the structures generated by a crystal structure prediction study of the unsubstituted chalcone. Hence the packing is dominated by the core, but since this has a wide variety of packings of similar stability, the packing changes required by the substituents produce a wide range of observed structures. There is little evidence for any crystal engineering principle of preferred chalcone scaffold packing beyond close packing of the specific molecule.

Some of the most important recent developments in the field relate to the application of machine learning (ML) techniques. ML can be used to reduce the computational cost associated with energy evaluations by using energies calculated from *e.g.* DFT as training sets to enable ML calculations of energies as a function of geometry: essentially ML potentials. More ambitiously ML can be used in direct structure prediction by using known structural databases as a training set as in the work of Ryan *et al.* (2018 ▸). The use of these methods will continue to grow, and they will be increasingly used together with the simulation methods discussed above. Another possible development will be the increased use of potential-based methods in energy evaluations, including, of course, ML potentials, but also high-quality ‘classical’ potentials based on analytical functions. There remain, of course, many challenges including modelling of disorder where there is a recent intriguing contribution from Dittrich (2021 ▸) who discusses the inclusion of restraints from theory in disorder refinement.

Over thirty years after the Maddox challenge, structure prediction is an exciting and rapidly developing field. **IUCrJ** welcomes articles in this and related areas.

## References

[bb98] Catlow, C. R. A. (2020). *IUCrJ*, **7**, 778–779. 10.1107/S2052252520011793 PMC7467169 32939267

[bb1] Catlow, C. R. A. & Price, G. D. (1990). *Nature*, **347**, 243–248.

[bb2] Catlow, C. R. A., Thomas, J. M., Parker, S. C. & Jefferson, D. A. (1982). *Nature*, **295**, 658–662.

[bb3] Dickson, R. C., Manning, T. D., Raj, E. S., Booth, J. C. S., Rosseinsky, M. J. & Dyer, M. S. (2022). *Phys. Chem. Chem. Phys.* **24**, 16374–16387. 10.1039/d2cp02180c 35762846

[bb4] Dittrich, B. (2021). *IUCrJ*, **8**, 305–318. 10.1107/S2052252521000531 PMC7924241 33708406

[bb5] Escatllar, A. M., Lazaukas, T., Woodley, S. M. & Bromley, S. T. (2019). *ACS Earth Space Chem.* **3**, 2390–2403. 10.1021/acsearthspacechem.9b00139 PMC7009040 32055761

[bb6] Foster, M. D., Simperler, A., Bell, R. G., Friedrichs, O. D., Paz, F. A. A. & Klinowski, J. (2004). *Nat. Mater.* **3**, 234–238. 10.1038/nmat1090 15048108

[bb7] Henson, N. J., Cheetham, A. K. & Gale, J. D. (1994). *Chem. Mater.* **6**, 1647–1650.

[bb8] Lazauskas, T., Sokol, A. A. & Woodley, S. M. (2017). *Nanoscale*, **9**, 3850–3864. 10.1039/c6nr09072a 28252128

[bb9] Oganov, A. R. (2018). *Faraday Discuss.* **211**, 643–660. 10.1039/c8fd90033g 30306975

[bb10] Pickard, C. J. & Needs, R. J. (2011). *J. Phys. Condens. Matter*, **23**, 053201. 10.1088/0953-8984/23/5/053201 21406903

[bb11] Price, L. S. & Price, S. L. (2022). *Cryst. Growth Des.* **22**, 1801–1816. 10.1021/acs.cgd.1c01381 PMC9097456 35571354

[bb12] Price, S. L. (2018). *Faraday Discuss.* **211**, 9–30. 10.1039/c8fd00121a 30051901

[bb99] Ryan, K., Lengyel, J. & Shatruk, M. (2018). *J. Am. Chem. Soc.* **140**, 10158–10168. 10.1021/jacs.8b03913 29874459

[bb13] Schön, J. C. & Jansen, M. (2009). *Int. J. Mater. Res.* **100**, 135–152.

[bb14] Treacy, M. M. J., Rivin, I., Balkovsky, E., Randall, K. H. & Foster, M. D. (2004). *Microporous Mesoporous Mater.* **74**, 121–132.

[bb15] Wells, A. F. (1954). *Acta Cryst.* **7**, 842–848.

[bb17] Woodley, S. M. & Catlow, R. (2008). *Nat. Mater.* **7**, 937–946. 10.1038/nmat2321 19029928

[bb18] Woodley, S. M., Day, G. M. & Catlow, R. (2020). *Philos. Trans. R. Soc. A.* **378**, 20190600. 10.1098/rsta.2019.0600 33100162

[bb16] Woodley, S. M. & Sokol, A. A. (2012). *Angew. Chem. Int. Ed.* **51**, 3752–3754. 10.1002/anie.201109030 22383381

[bb19] Yang, S. & Day, G. M. (2022). *Commun. Chem.* **5**, 86–98. 10.1038/s42004-022-00705-4 PMC9814927 36697680

